# NR4A1 inhibits the epithelial–mesenchymal transition of hepatic stellate cells: Involvement of TGF-β–Smad2/3/4–ZEB signaling

**DOI:** 10.1515/biol-2022-0047

**Published:** 2022-05-04

**Authors:** Qian Huang, Jingying Xu, Yanyan Ge, Yue Shi, Fei Wang, Mingli Zhu

**Affiliations:** Department of Infectious Diseases, Hangzhou Xixi Hospital, Zhejiang University of Traditional Chinese Medicine, Hangzhou, 310023, China; Department of Internal Medicine, Hangzhou Third People’s Hospital, Zhejiang University of Traditional Chinese Medicine, Hangzhou, 310009, China; Department of Laboratory Medicine, Hangzhou Xixi Hospital, Zhejiang University of Traditional Chinese Medicine, Hangzhou, 310023, China

**Keywords:** liver fibrosis, nuclear receptor 4a1, epithelial–mesenchymal transition

## Abstract

This study aimed to examine whether nuclear receptor 4a1 (NR4A1) is involved in inhibiting hepatic stellate cell (HSC) activation and liver fibrosis through the epithelial–mesenchymal transition (EMT). HSC-T6 cells were divided into the control group, the acetaldehyde (200 μM, an EMT activator) group, and the NR4A1 activation group (Cytosporone B; 1 μM). The expression levels of the epithelial marker E-cadherin, the mesenchymal markers fibronectin (FN), vimentin, smooth muscle alpha-actin (α-SMA), and fibroblast-specific protein 1 (FSP-1), and the components of the transforming growth factor (TGF)-β pathway were detected by real-time polymerase chain reaction and western blotting. Compared with the control group, E-cadherin in the acetaldehyde group was downregulated, whereas FN, FSP-1, vimentin, α-SMA, and COL1A1/COL1A2 were upregulated (*P* < 0.05). Compared with the acetaldehyde group, NR4A1 agonist upregulated E-cadherin and downregulated FN, FSP-1, vimentin, α-SMA, and COL1A1/COL1A2 (*P* < 0.05). After acetaldehyde stimulation, TGF-β, Smad2/3/4, and zinc finger E-box-binding homeobox (ZEB) were upregulated, while Smad7 mRNA levels were downregulated (all *P* < 0.05). Compared with acetaldehyde alone, NR4A1 agonist increased Smad7 mRNA levels and reduced TGF-β, Smad2/3/4, and ZEB mRNA levels (all *P* < 0.05). NR4A1 activation suppresses acetaldehyde-induced EMT, as shown by epithelial and mesenchymal marker expression. The inhibition of the TGF-β–Smad2/3/4–ZEB signaling during HSC activation might be involved.

## 1 Introduction

Liver fibrosis is a wound-healing response to chronic liver injury caused by viral hepatitis, alcohol, metabolic diseases, autoimmune conditions, and cholestatic liver diseases. The most common etiologies are alcoholic liver disease, non-alcoholic fatty liver disease, chronic viral hepatitis, genetic conditions (*α* − 1 antitrypsin deficiency, hereditary hemochromatosis, and Wilson disease), and autoimmune diseases (primary biliary cirrhosis, primary sclerosing cholangitis, and autoimmune hepatitis), and drugs. Sustained liver damage leads to fibrosis, liver failure, and even hepatocellular carcinoma [1,2]. In the United States, the prevalence of chronic liver diseases was 1.8% in 2017, with 12.8 deaths per 100,000 population [3,4].

The epithelial–mesenchymal transition (EMT) is a transition of epithelial cells to a mesenchymal state, which is a reversible process [5]. The EMT plays an essential role in tissue development, wound healing, fibrosis, and cancer progression [6,7,8]. Liver fibrosis is caused by excess extracellular matrix (ECM) production by myofibroblasts [9]. Activation of hepatic stellate cells (HSCs) is a key event in the formation of liver fibrosis since it is the major source of myofibroblasts [10]. Several studies indicated that HSCs undergo EMT during their activation [7,11,12,13] to participate in liver fibrosis [14,15,16]. The main pathways involved in the EMT in liver fibrosis are the Hedgehog signaling pathway, transforming growth factor (TGF)-β signaling pathway, Notch signaling, and extracellular signal-regulated kinase (ERK) signaling pathway [7,17]. Excessive Hedgehog activation after liver injury participates in EMT and liver fibrosis [7,17]. TGF-β signaling is involved in ECM and collagen production by HSCs [7,17]. The ERK pathway plays a crucial role in cell growth and differentiation and represses EMT [17]. The Notch pathway is involved in cell differentiation [7]. Importantly, inhibition of the EMT of HSCs can suppress the activation of the HSCs, thereby alleviating the progression of hepatic fibrosis [11,18,19]. Hence, inhibition of the EMT is a promising strategy for reversing fibrosis.

Nuclear receptor 4a1 (NR4A1, also known as Nur77, TR3, or NGFIB) is a member of the NR4A family of nuclear orphan receptors. NR4A1 plays diverse and important regulatory roles in glucose and lipid metabolism and inflammatory responses [20,21]. NR4A1 is involved in the EMT in tumor metastasis and migration [22,23,24]. The loss of NR4A1 inhibits TGF-β-induced EMT and metastasis [22]. Furthermore, Palumbo-Zerr et al. [25] demonstrated that NR4A1 inhibits TGF-β signaling and can suppress experimental lung and liver fibrosis.

Additional recent studies have also demonstrated that NR4A1 inhibits TGF-β signaling [26,27,28]. Therefore, NR4A1 might represent a promising antifibrotic target, but since the EMT mechanisms associated with liver fibrosis and those associated with cancer might be different, it remains unclear whether NR4A1 inhibits HSC activation and liver fibrosis by modulating the EMT. This study aimed to examine whether NR4A1 is involved in inhibiting HSC activation and liver fibrosis through the EMT.

## 2 Materials and methods

### 2.1 Cells

The HSC-T6 cells were purchased from Shanghai Tongpai Technology Co., Ltd. (Shanghai, China). After synchronization, the HSC-T6 cells were divided into three groups: the control group (HSC-T6 cells plus an equal volume of medium), acetaldehyde (MACKLIN, Shanghai, China; purity: ≥99%), the treatment group (HSC-T6 cells plus acetaldehyde to a final concentration of 200 μM), and the NR4A1 activation group (HSC-T6 cells plus acetaldehyde to a final concentration of 200 μM and Cytosporone B [Csn-B; Santa Cruz Biotechnology, Inc., Dallas, TX, USA; purity: ≥98%] to a final concentration of 1 μM).

### 2.2 Quantitative real-time PCR

Total RNA was extracted from the cells using TRIzol (MiniBEST Universal RNA Extraction Kit; Takara Bio, Otsu, Japan). Gene expression was measured by the quantitative real-time polymerase chain reaction (qRT-PCR) using the SYBR Green Real-time PCR Master Mix (Takara, Otsu, Japan) performed under standard conditions with an ABI 7900 Sequence Detection System (Applied Biosystems, Foster City, CA, USA). All primers were from Takara. The primer sequences are listed in Table 1.

**Table 1:** Primer sequences for real-time PCR

Gene	Primer sequences (5′–3″)	Product size (bp)	Designed *T*_m_ (°C)
FSP-1	Forward: ATGTAATAGTGTCCACCTTCC	181	54.71
	Reverse: ACTTCATTGTCCCTGTTGCT		57.34
a-SMA	Forward: GGAGAAGCCCAGCCAGTCGC	115	65.58
	Reverse: CCCGCCTTACAGAGCCCGGA		66.26
E-cadherin	Forward: TGTTGATAGCGTGCCCTTTG	100	58.84
	Reverse: GTTCCGATTGCTTGCCTTTT		57.56
FN	Forward: GGATCCCCTCCCAGAGAAGT	188	60.03
	Reverse: GGGTGTGGAAGGGTAACCAG		59.96
COL1A2	Forward: AGGGTGTTCAAGGTGGCAAA	166	60.03
	Reverse: CCACGTTCTCCTCTTGGACC		60.04
COL1A1	Forward: AAAACCACCAAGACCTCCCG	141	60.18
	Reverse: GGTGGGAGGGAACCAGATTG		60.03
Vimentin	Forward: ACCGCTTCGCCAACTACATC	138	60.74
	Reverse: GCAACTCCCTCATCTCCTCCT		60.97
GADPH	Forward: TCTCTGCTCCTCCCTGTTCT	95	59.59
	Reverse: ATCCGTTCACACCGACCTTC		60.04
Smad2	Forward: GCCGCCCGAAGGGTAGAT	164	61.55
	Reverse: TTCTGTTCTCCACCACCTGC		59.89
Smad3	Forward: ATACGGATGTTCAAGTGTTCG	242	56.38
	Reverse: ACTGGGTCCTCTTTGGTTTT		56.85
Smad4	Forward: ATCCACCAAGTAATCGCGCA	252	60.11
	Reverse: AGGTGGTAGTGCTGTTATGGTG		60.03
Smad7	Forward: GTGGCATACTGGGAGGAGAA	309	58.80
	Reverse: GATGGAGAAACCAGGGAACA		57.12
ZEB	Forward: CCAAAGCAACAGGGAGAGTTAC	397	59.19
	Reverse: CTTGTCTTTCATCCTGGTTTCC		57.23
TGF-β	Forward: GAGGCGGTGCTCGCTTTGTA	211	60.00
	Reverse: GCACTGCTTCCCGAATGTCTG		57.14

#### 2.2.1 Genomic DNA removal from total RNA

The genomic DNA was removed using the following components: 5× gDNA Eraser buffer 1 (2 μL), gDNA Eraser (1 μL), and total RNA (2 μL). The reaction conditions were as follows: 42°C, 2 min; 4°C, 5 min.

#### 2.2.2 Calculation of the gene expression

First, we calculated the average Ct value of the sample, and then, the ∆Ct of the target gene in the sample. The relative value of the target gene in the sample to the internal reference gene was calculated. Afterward, the ∆Ct of a certain gene was calculated relative to the reference sample group and multiple relationships.

#### 2.2.3 Reference gene selection

GAPDH has been used as a stable reference gene selected as a housekeeping conserved gene according to previous studies [29,30,31,32,33,34].

### 2.3 Western blot

Protein lysate was obtained from the cultured cells using the RIPA lysis buffer (Solarbio Corporation, Beijing, China). The protein concentration was measured with a bicinchoninic acid assay. Proteins were separated by 10% sodium dodecyl sulfate polyacrylamide gel electrophoresis and transferred onto polyvinylidene fluoride membranes. The membrane was first incubated with rabbit anti-rat primary antibodies, such as E-cadherin (1:2,000; no. 20874-1-AP), fibronectin (FN; 1:3,000; no. 15613-1-AP), vimentin (1:2,000; no. 10366-1-AP), Smad2 (1:2,000; no. 12570-1-AP), Smad4 (1:2,000; no. 51144-1-AP), zinc-finger-enhancer-binding protein 1 (ZEB; 1:2,000; no. 21544-1-AP) (all from Proteintech Group Inc., Chicago, IL, USA), Smad3 (1:2,000, no. AF6362; Affinity BIO, Scoresby, Australia), Smad7 (1:2,000; no. AF5147; Affinity BIO), and β-actin (1:5,000; no. Ab8226; Abcam, Cambridge, UK), and then incubated with goat anti-rabbit horseradish peroxidase-conjugated-labeled secondary antibodies (1:5,000, # A0208, Beyotime Institute of Biotechnology, Haimen, China) for 1 h at room temperature. The blots were detected using enhanced chemiluminescence.

### 2.4 Statistical analysis

The data are presented as mean ± standard deviation and were analyzed using a one-way analysis of variance with Fisher’s least significant difference post hoc test. *P*-values <0.05 were considered statistically significant (*P < 0.05 and **P < 0.01).

## 3 Results

### 3.1 NR4A1 regulates the expression of EMT-related genes to prevent EMT in HSC-T6 cells

**Figure 1:**
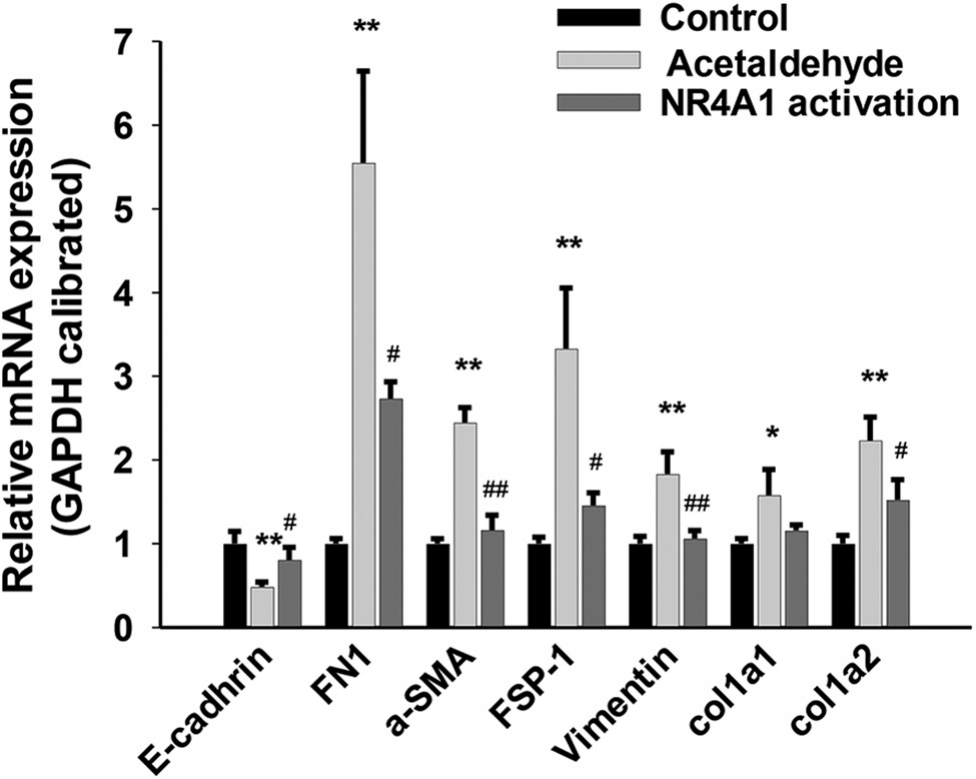
Effects of NR4A1 on EMT-related genes in HSC-T6 cells. The mRNA levels of E-cadherin in the acetaldehyde group were significantly downregulated, whereas FN, FSP-1, vimentin, α-SMA, and COL1A1/COL1A2 were significantly upregulated. The mRNA levels of E-cadherin in the NR4A1 activation group were significantly upregulated, while FN, FSP-1, vimentin, α-SMA, and COL1A1/COL1A2 were significantly downregulated. The mRNAs were analyzed by qRT-PCR analysis. *P < 0.05, **P < 0.01 vs control, ^#^ *P* < 0.05, ^##^ *P* < 0.01 vs acetaldehyde. *n* = 3/group.

**Figure 2:**
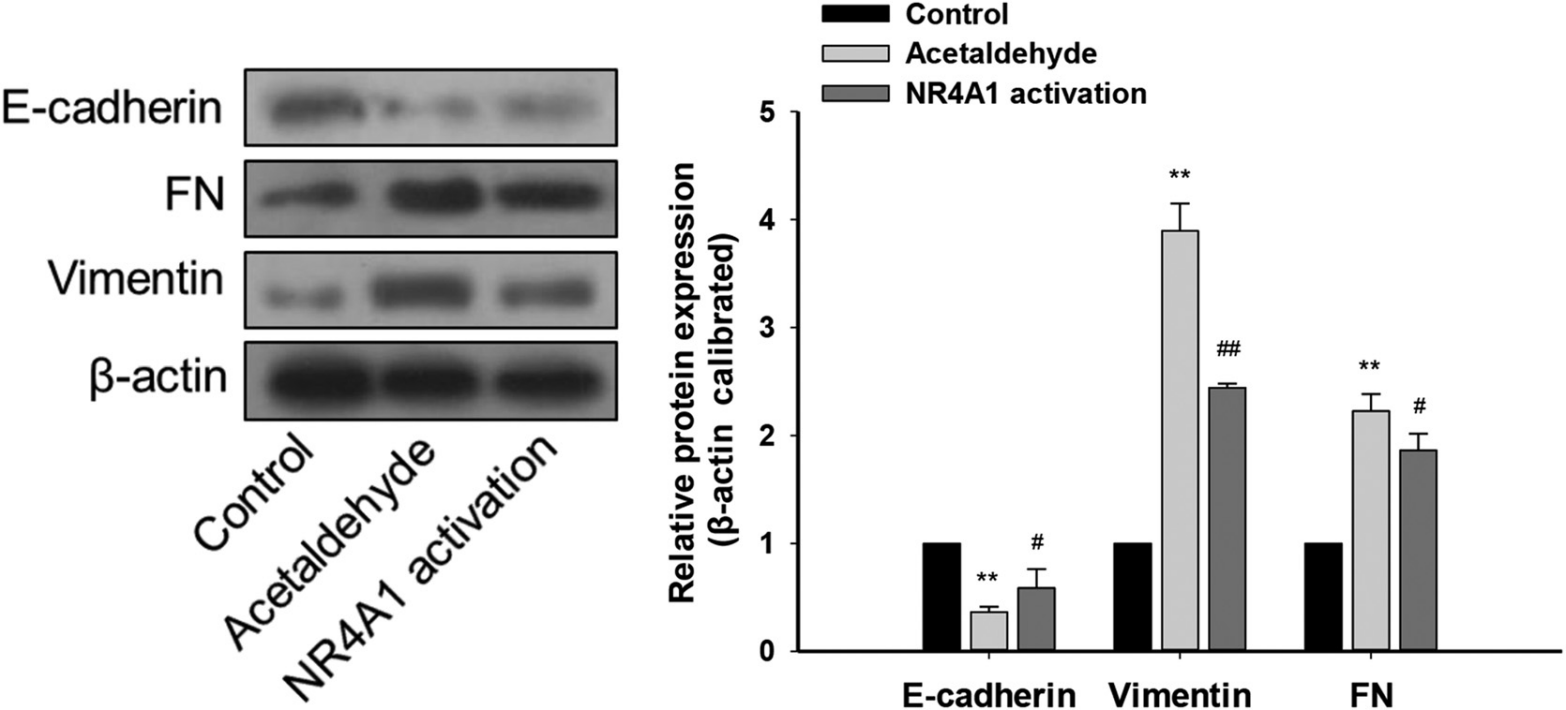
Effects of NR4A1 on EMT-related proteins in HSC-T6 cells. Protein levels of E-cadherin (epithelial marker) in the acetaldehyde group were downregulated, while those of FN and vimentin (mesenchymal markers) were upregulated. The protein levels of E-cadherin were upregulated in the NR4A1 activation group, while those of FN and vimentin were downregulated. The proteins were analyzed by western blotting. β-Actin was used as an internal control. **P < 0.01 vs control, ^#^ *P* < 0.05, ^##^ *P* < 0.01 vs acetaldehyde. *n* = 3/group.

To explore the regulatory effects of NR4A1 on the EMT of HSC-T6 cells, we first used acetaldehyde to stimulate HSC-T6 cells and measured the changes in the expression of EMT-related genes. Compared with the control group, the mRNA levels of E-cadherin in the acetaldehyde group were significantly downregulated, whereas those of FN, fibroblast-specific protein 1 (FSP-1), and vimentin were significantly upregulated. In addition, the mRNA levels of HSC activation markers, including smooth muscle alpha-actin (α-SMA) and COL1A1/COL1A2, were significantly upregulated in the acetaldehyde group compared with the control group. When the NR4A1 agonist (Csn-B) was used with acetaldehyde, compared with the acetaldehyde group, the mRNA levels of E-cadherin in the NR4A1 activation group were significantly upregulated, while those of FN, FSP-1, vimentin, α-SMA, and collagen genes (COL1A1/COL1A2) were significantly downregulated (Figure 1). Similar changes were observed at the protein level (Figure 2). These results indicated that the acetaldehyde model induces EMT in HSC-T6 cells and that NR4A1 is involved in regulating the expression of EMT-related genes, probably preventing EMT in the cells.

### 3.2 NR4A1 possibly inhibits EMT through the TGF-β–Smad–ZEB signaling pathway

**Figure 3:**
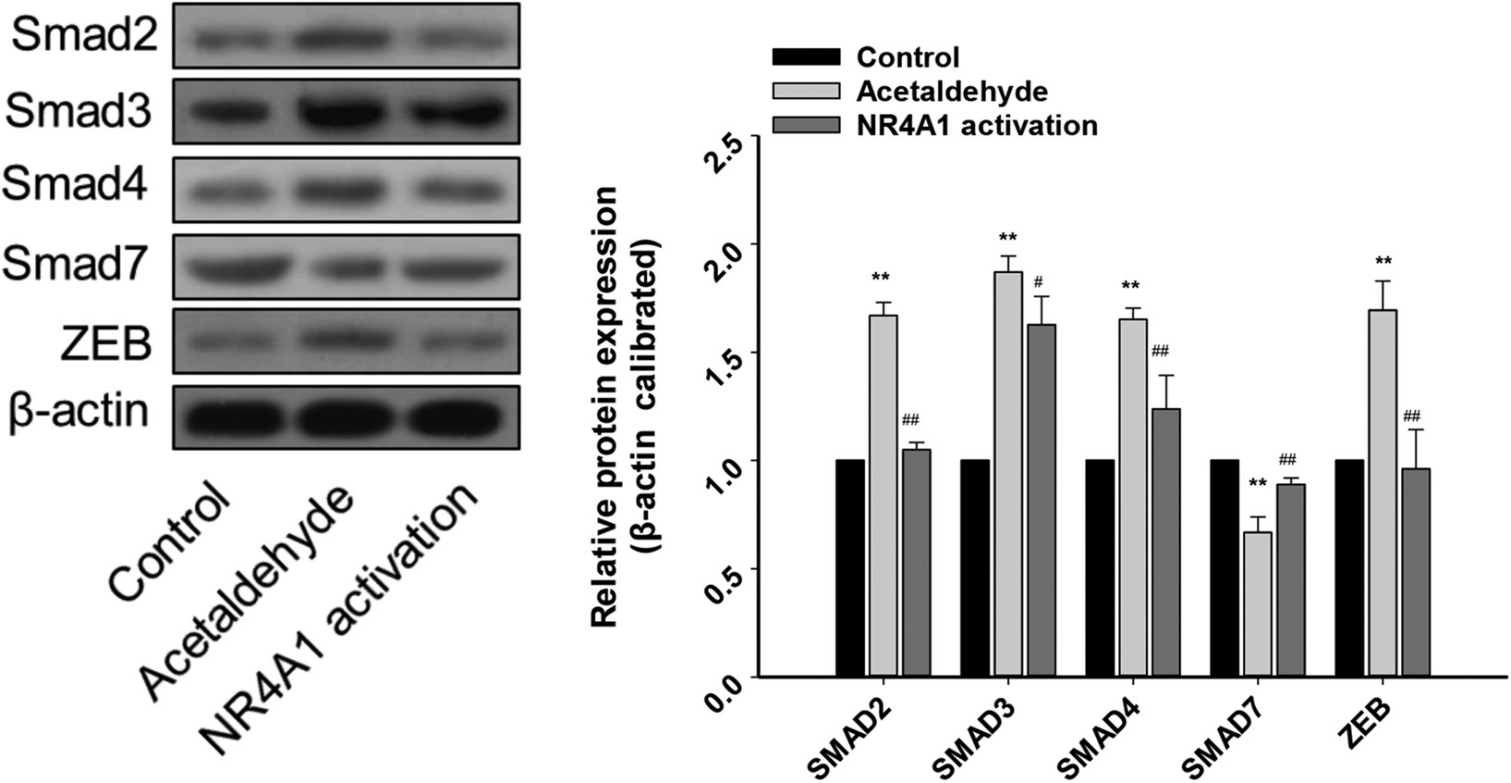
Effects of NR4A1 on the protein levels of Smad2/3/4, Smad 7, and ZEB. Protein levels of Smad2/3/4 and ZEB in the acetaldehyde group were upregulated, while that of Smad 7 was downregulated. The expression of these proteins was reversed in the NR4A1 activation group. The proteins were analyzed by western blotting. β-Actin was used as an internal control. *P < 0.05, **P < 0.01 vs control, ^#^ *P* < 0.05, ^##^ *P* < 0.01 vs acetaldehyde. *n* = 3/group.

**Figure 4:**
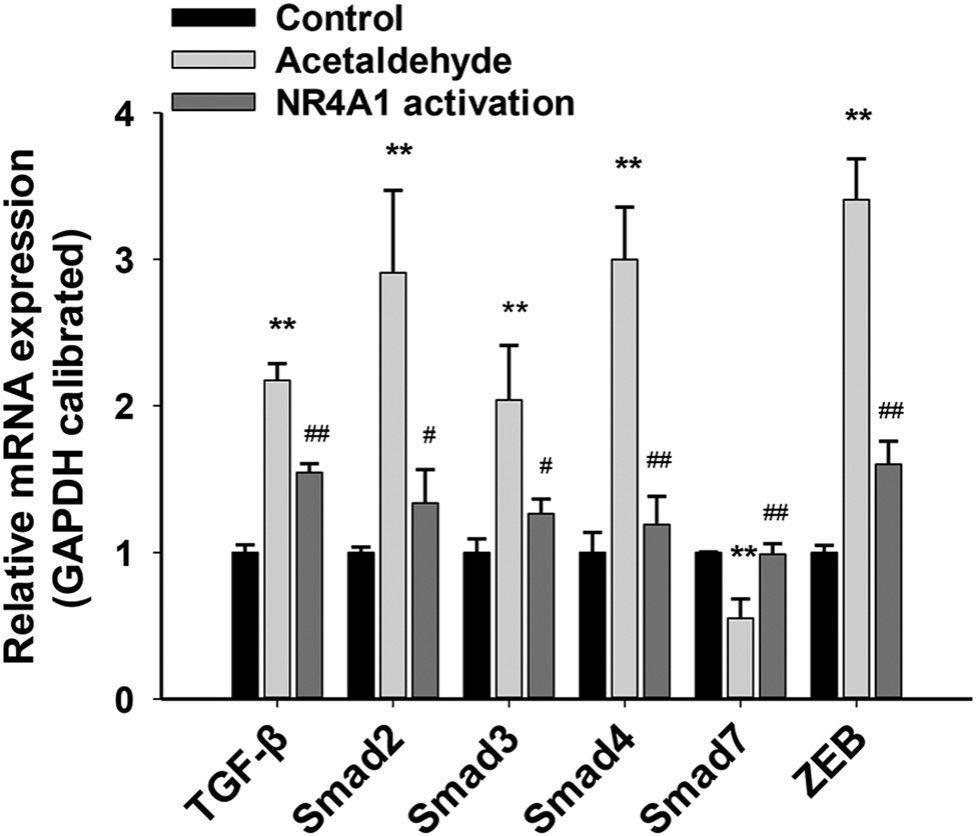
mRNA levels of the components of the TGF-β–Smad–ZEB signal pathway in HSC-T6 cells. The mRNA levels of TGF-β, Smad2/3/4, and ZEB were significantly upregulated, while Smad7 mRNA levels were significantly downregulated. The mRNA levels of Smad7 in the NR4A1 activation group were significantly upregulated, while those of TGF-β, Smad2/3/4, and ZEB were significantly downregulated. *P < 0.05, **P < 0.01 vs control, ^#^ *P* < 0.05, ^##^ *P* < 0.01 vs acetaldehyde. *n* = 3/group.

The TGF-β–Smad–ZEB signaling pathway is involved in the EMT [6]. Therefore, we explored whether NR4A1 activation inhibits EMT by regulating the TGF-β–Smad–ZEB signaling pathway. After acetaldehyde was used to stimulate HSC-T6 cells, TGF-β, Smad2/3/4, and ZEB mRNA levels were significantly upregulated, while Smad7 mRNA levels were significantly downregulated. Compared to these levels following acetaldehyde alone, acetaldehyde combined with an NR4A1 agonist (Csn-B) resulted in increased Smad7 mRNA levels and reduced TGF-β, Smad2/3/4, and ZEB mRNA levels (Figure 3). Similar changes were also observed at the protein level (Figure 4). Collectively, these results suggest that during acetaldehyde-induced HSC activation and EMT, NR4A1 activation suppresses the EMT, at least in part, by inhibiting the TGF-β–Smad–ZEB signaling pathway.

## 4 Discussion

Activation of HSCs is a key event in liver fibrosis [10]. HSCs undergo EMT during activation [7,11,12,13]. NR4A1 inhibits TGF-β signaling, which can suppress experimental lung and liver fibrosis [25], but whether NR4A1 inhibits HSC activation and liver fibrosis through the EMT is unknown. Therefore, this study aimed to examine whether NR4A1 is involved in inhibiting HSC activation and liver fibrosis through the EMT. The results suggest that NR4A1 activation suppresses acetaldehyde-induced EMT in HSCs, as shown by increased epithelial and decreased mesenchymal marker expression. The inhibition of the TGF-β–Smad2/3/4–ZEB signaling during HSC activation might be involved.

Liver fibrosis is a wound-healing response to various liver damage forms, such as hepatitis, alcohol, drugs, metabolic diseases, biliary injury, and toxins. Importantly, HSC activation is a key event in liver fibrosis [10], and wounding and repair are dynamic processes that include matrix synthesis, deposition, and degradation [35]. HSC activation undergoes EMT-like changes and participates in fibrogenesis [11,12]. Consistent with these previous studies, the present study indicated that the acetaldehyde-induced activation of HSCs upregulated the expression levels of FN, FSP-1, vimentin, and α-SMA (all myofibroblastic markers) while downregulating those of E-cadherin (an epithelial marker). Hence, these findings confirm that HSC activation involves the EMT.

Several previous studies have shown that NR4A1 can promote tumor cells’ EMT [22,23,24]. Still, it remains unclear whether the role of NR4A1 in EMT in cancer is the same as in EMT in liver fibrosis and HSC activation. Csn-B is an NR4A1 agonist that enhances the transcriptional activity of NR4A1. The present study showed that Csn-B upregulated the expression levels of epithelial markers and decreased the expression levels of mesenchymal markers in acetaldehyde-induced EMT in HSCs, compared to these levels in the presence of acetaldehyde alone. These findings suggest that NR4A1 activation suppressed the EMT during acetaldehyde-induced activation of HSCs. It is contrary to the findings in tumor cells, which is not surprising since the pathogeneses and cellular characteristics of tumors and fibrosis exhibit different characteristics. In the present study, the mechanism of NR4A1-mediated inhibition of the EMT of HSCs was explored. Palumbo-Zerr et al. [25] found that NR4A1 is an endogenous inhibitor of TGF-β signaling and inhibits skin, lung, liver, and kidney fibrosis. TGF-β signaling has also been identified as one of the predominant inducers of the EMT [36,37], and the inhibition of TβRI activity can block TGFβ‑induced EMT. TGF-β binds to TβR receptors to form a complex, activating Smad2/Smad3 to interact with Smad4 to form trimeric Smad complexes. The TGFβ/Smad signaling pathway activates the expression of EMT transcription factors and initiates the EMT [6,38]. Smad complexes interact with ZEB1 and ZEB2 to mediate TGFβ‑regulated gene expression. ZEB is one of the key transcription factors of the EMT, and its functions are finely regulated at the transcriptional, translational, and post-translational levels. ZEB expression is activated early during the EMT and plays a central role in developing both fibrosis and cancer. The TGF–Smad–ZEB pathway is involved in the EMT [6]. In the present study, we found that acetaldehyde-mediated stimulation of HSCs significantly upregulated TGF-β, Smad2/3/4, and ZEB levels and significantly downregulated Smad7 levels. Furthermore, NR4A1 activation in the presence of acetaldehyde resulted in higher Smad7 levels and lower TGF-β, Smad 2/3/4, and ZEB expression levels than acetaldehyde treatment alone. Previous studies confirmed that Smad7 inhibits TGF-β signaling [39]. The present study illustrates that NR4A1 can suppress EMT by inhibiting TGF-β–Smad2/3/4–ZEB signaling. NR4A1 might negatively regulate TGF-β signaling, at least in part, by promoting SMAD7 expression.

NR4A1 activates TGF signaling and promotes EMT in tumor cells [22,23], whereas the present study in HSCs differs from those in tumor cells. Interestingly, NR4A1 exhibits both tumor-suppressive and pro-oncogenic effects in cancer development [40,41,42]. NR4A1 translocation from the nucleus to the cytoplasm in colon cancer cells may initiate apoptotic cascades [43]. In contrast, NR4A1 exhibits anti-apoptotic effects when it is not exported from the nucleus [41,44]. TGF-mediated induction of the EMT is dependent on the nuclear export of NRA41 in breast cancer cells, and NR4A1 antagonists inhibit the nuclear export of NR4A1 and thereby block the TGF-induced EMT [23]. NR4A1 phosphorylation decreases the transcriptional activity of NR4A1, and pNR4A1 is strongly associated with hepatic/lung fibrosis and is mainly located in the cytoplasm, whereas pan-NR4A1 localizes in both nuclear and cytoplasmic compartments [25]. Collectively, these studies suggest that the effects of NR4A1 depend on its subcellular localization and the cell type in which it is signaling.

This study has limitations. The subcellular localization and expression of NRA4A1 were not examined, which could be an added benefit for this study. Thus, this should be tested in future experiments. In addition, the pathways involved in EMT and fibrosis were examined superficially. Only an agonist of NRA4A1 was used, and future studies should also use an antagonist. In addition, agonists/antagonists and silencing/overexpression of TGF-β and other proteins involved in that pathway should be used to determine the contribution of the TGF-β pathway in the EMT in liver fibrosis. Nonetheless, this study only used GAPDH as a reference gene due to the fact that several studies used GAPDH as an internal reference [29–34]. However, it is recommended to use at least two reference genes to obtain more reliable results, and therefore, other reference genes will be considered for future studies.

In summary, this study indicates that NR4A1 suppresses the EMT during acetaldehyde-induced HSC activation. NR4A1-mediated inhibition of the EMT of HSCs is involved in the suppression of TGF-β–SMAD2/3/4–ZEB signaling and increased SMAD7 expression, but confirmation is needed. Hence, the findings suggest that NR4A1 plays an important role during HSC activation and that NR4A1 might be a promising therapeutic target for treating liver fibrosis.
